# Adverse Childhood Experiences and Psychological Well-Being in Chinese College Students: Mediation Effect of Mindfulness

**DOI:** 10.3390/ijerph18041636

**Published:** 2021-02-09

**Authors:** Chien-Chung Huang, Yuanfa Tan, Shannon P. Cheung, Hongwei Hu

**Affiliations:** 1 School of Social Work, Rutgers, The State University of New Jersey, New Brunswick, NJ 08901, USA; huangc@ssw.rutgers.edu (C.-C.H.); scheung@ssw.rutgers.edu (S.P.C.); 2 Research Institute of Social Development, Southwestern University of Finance and Economics, Chengdu 611130, China; 3 School of Public Administration and Policy, Renmin University of China, Beijing 100872, China; whuhhw@126.com

**Keywords:** psychological well-being, mindfulness, adverse childhood experiences, students, China, college

## Abstract

Literature on the antecedents of psychological well-being (PWB) has found that adverse childhood experiences (ACEs) and mindfulness are associated with PWB; less is known, however, about the role of mindfulness, a type of emotional and self-regulation, in the pathway between ACEs and PWB. This study used data from 1871 college students across China to examine the relation between ACEs and PWB, and whether the relation was mediated by mindfulness. The findings from structural equation modelling indicate a statistically significant negative association between ACEs and PWB, while mindfulness was strongly and positively associated with PWB. The effect of ACEs on PWB was reduced once mindfulness was controlled for in the analysis. This provides evidence that mindfulness was able to partially mediate the effects of negative life experiences on psychological well-being. This calls for mindfulness interventions targeted toward students with a history of ACEs to buffer the effects of ACEs on PWB.

## 1. Introduction

Psychological well-being (PWB) is an important indicator of life outcomes and has been found to be associated with health, quality of life, development, and success [1,2]. High PWB has been associated with better physical and mental health, as well as greater life satisfaction [3,4]. PWB is also related to higher levels of employment, income, work performance, social support, and development in later life [1,5].

Given the importance of PWB, many studies have attempted to understand the factors associated with PWB and found that life experience and mindfulness were important ones [6,7,8]. Life experiences such as adverse childhood experiences (ACEs) were associated with greater mental health problems and lower PWB [9,10,11]. For example, Merrick and colleague found that ACEs were associated with low PWB such as high mental health problems and risky behaviors in adulthood [10]. Meanwhile, mindfulness is associated with low psychological distress and high PWB [7,12]. Parto and Besharat showed that mindfulness had a strong and negative effect on psychological distress and a strong and positive effect on PWB [12]. Although literature has found that ACEs and mindfulness are associated with PWB, less is known on whether mindfulness, an indicator of emotional and self-regulation, mediates the effects of ACEs on PWB and, if so, the extent to which it mediates these effects.

The extant literature on ACEs has focused on children and middle-aged adults and, largely, Western samples but less so on the emerging adult population and those individuals who come from non-Western cultures [8,13]. Because ACEs are operationalized as events that occur prior to individuals’ 18th birthday, the most immediate time period wherein scholars may observe their effects on PWB is emerging adulthood, the life stage that takes place when individuals are between 19 and 24 years old [14,15]. The college years have been found to be a particularly important time for lifespan development [14,15,16,17,18,19], as this period is characterized by increasing independence and responsibility [14,15]. The study of factors that increase PWB are important to shed light on the human capacity to thrive in the face of challenging life circumstances, including those that may occur during a critical transitory period between adolescence and adulthood [4,14,15]. Thus, this study aims to use a non-Western sample to understand the relation between ACEs and PWB and whether it is mediated by mindfulness in college students in China, one of the largest populations in the non-Western world.

## 2. Conceptual Framework and Hypotheses

### 2.1. Psychological Well-Being (PWB)

PWB contains multiple dimensions, including self-acceptance, positive relations with others, autonomy, environmental mastery, purpose in life, and personal growth [20]. Self-acceptance denotes a positive attitude toward the self, including the acceptance of the self and positive feelings about the past experiences. Positive relations with others refer to an individual’s perception that they have warm, satisfying, and trusting relationships with others and that they are capable of strong empathy, affection, and intimacy. Autonomy is defined by an individual’s self-determination and independence, as well as their ability to resist social pressures to think and act in certain ways. Environmental mastery means they have a sense of mastery and competence in managing their environment and are able to choose or create contexts suitable to personal needs and values. Purpose in life indicates that a student is goal-driven and has a sense of direction in life. Finally, personal growth indicates a feeling of continued development and seeing themselves as growing and expanding [21].

The multiple dimensions of PWB are crucial for well-being and fundamentally anchored in how individuals navigate their way through the challenges of life [4]. The integration of mental health, clinical, and life-span development theories point to the converging aspects of positive psychological functioning, which includes the aforementioned dimensions [20]. Empirical studies have supported that the multiple-dimensional PWB measure is a comprehensive indicator of psychological functioning and future life outcomes [3,4].

Many empirical studies on PWB have identified various factors associated with PWB [4]. Some of the factors related to the study of PWB include personality traits, self-regulation, and life experience [4,21,22,23,24,25]. Personality traits appear to be one of the most influential factors, explaining about 20–30% of variance in psychological or subjective well-being [23,25]. For example, teenage females who were more extraverted had greater PWB in midlife, contrasted by their peers with high neuroticism and lower PWB later in life [23]. Self-regulation is the ability to monitor and manage emotions, thoughts, and behaviors that, over time, help to maximize adaptive adjustment [26,27]. Capacity to self-regulate can produce the means to effectively confront and adapt to life’s challenges and increases PWB in individuals [28,29]. Studies have shown that mindfulness is strongly related to personality traits and self-regulation [30,31]. Giluk’s meta-analysis found that mindfulness displayed appreciable relationships with all big five personality traits (neuroticism, extraversion, openness to experience, agreeableness, and conscientiousness). Mindfulness showed a large and negative association with neuroticism but positive association with the other four traits [30]. Mindfulness has also been found to be associated with high self-regulation, low psychological distress, and high PWB [7,12,31]. For example, Kaunhoven and Dorjee demonstrated that mindfulness was associated with brain networks that underlie self-regulatory abilities [31]. Meanwhile, ACEs have been found to have negative effects on mental health, life satisfaction, and PWB [11,12,13]. For example, using 6323 participants from three waves of the Midlife Development in the United States study from 1995 to 2014, Mosley-Johnson and colleagues found that higher ACE counts were associated with lower life satisfaction compared to those with no ACEs [8]. In short, literature has found that ACEs are negatively associated with PWB, while mindfulness is positively associated with PWB. However, whether mindfulness mediates these effects—and the extent to which it does—is yet to be examined.

### 2.2. ACEs and Mindfulness

Studies have shown that, regardless of the geographic location, almost two-thirds of youth have experienced a significant adverse event [32]. ACEs, including abuse (psychological, physical, or sexual), neglect, household challenges such as violence perpetrated against mother and cohabitation with individuals who use substances or have mental illness or incarceration history [33,34], from the first 18 years of life have been linked to a plethora of social and health issues in later childhood [15], adolescence [35,36,37], and adulthood [38,39,40].

Mindfulness is a state of consciousness in which individuals purposefully pay attention to the present moment and react in a non-judgmental manner [12,31]. Mindful attention, a key component of mindfulness, regulates individuals’ attention, brings their awareness to the present moment, and makes individuals alert to what is occurring in the here-and-now. Another key component of mindfulness is mindful metacognition or decentering, wherein individuals detach from the monitoring of thoughts and feelings about the ongoing events. During mindful metacognition, individuals mentally step back from their feelings and thoughts to remain non-judgmental [41,42]. Mindful attention and metacognition are highly correlated (*r* = 0.44, *p* < 0.001) [41].

### 2.3. Conceptual Framework: ACEs, Mindfulness, and PWB

The conceptual framework of this study is based on trauma theory [43]. Trauma theory posits that traumatic experiences, including those events that are considered ACEs, can impede the psychological well-being of individuals through the development of three symptom clusters: hyperarousal, constriction, and intrusion. Hyperarousal, a key symptom of posttraumatic stress disorder (PTSD), occurs when an individual’s sympathetic nervous system is activated by a traumatic memory. The chronicity of hyperarousal reproduces a prolonged state of self-protective vigilance that is difficult to turn off or regulate. Often, in response, traumatized individuals experience another symptom cluster, constriction, wherein they may become physiologically, emotionally, and cognitively unresponsive to stimuli. While constriction can functionally help individuals avoid painful trauma-related responses, intrusion may break through, forcing the survivor to relive the trauma through fragmentary images and vivid sensations of the original experience, notably, in the form of nightmares. As a result, traumatic experiences overwhelm the victims, disrupt their inner schemas about safety and trust, and strip them of control, connection, and meaning in life. Accordingly, traumatized individuals are highly sensitive to possible threats of danger, feeling as though danger may occur at any time. They are likely to dissociate from present situations, disrupting the ability to engage in mindfulness, and impeding their PWB in the long run [7,31,43].

Extensive empirical studies have provided support for this theoretical framework and relations between ACEs, mindfulness, and PWB. First, with respect to the studies on ACEs and PWB, ACEs are traumatic events that can have profound consequences throughout one’s lifetime [44,45] and are associated with greater incidence of mental health problems and lower life satisfaction and PWB [8,46]. For example, Anda and colleagues conducted a study on 17,337 adults and found that higher ACE scores significantly increased the risks of affective, somatic, and aggression-related outcomes. Moreover, the mean number of comorbid outcomes tripled across the range of ACE scores [46].

Second, studies on mindfulness and PWB and other life outcomes have shown that mindfulness promotes students’ academic performance [47,48], advances social and emotional competence [49,50,51] and alleviates emotional and behavioral problems [51,52]. Researchers have identified mindfulness as a potential promotive factor of PWB [7,12]. People with higher levels of mindfulness are prone to having better self and emotional regulation so that they are more capable of recognizing, managing, and resolving emotions and life problems, as well as of making decisions that are beneficial to their PWB [7,53].

Third, regarding studies on the effects of ACEs on mindfulness and related outcomes, ACEs have been shown to be associated with poorer mental health, reduced stress reactivity, and increased depressed affect and impulsive behavior [10,46,54]. For example, Lovallo used a cohort of 426 healthy young adults from the Oklahoma Family Health Patterns Project and examined the effects of ACEs on psychosocial stress and health outcomes. The results supported a pathway from early life adversity to low stress reactivity that shapes a basis for risky behaviors and poor health outcomes [54]. Thus, it is likely that ACEs may lead to lower PWB and poorer health outcomes by way of reduced mindfulness, a construct that is strongly related to emotional and self-regulation capacity. Indeed, studies have shown that ACEs are negatively related to mindfulness [55,56] and may serve as a mediator between ACEs and PWB or other related health outcomes [57,58,59]. For example, using 743 patients aged between 18 and 30, Nagel and colleagues found that ACEs were significantly and negatively associated with dispositional mindfulness (*p* < 0.001), a trait that allows one to be aware of the present moment. Dispositional mindfulness, measured by the Revised Cognitive and Affective Mindfulness Scale (CAMS-R), ranged 12–48 and averaged 35.2 for patients without ACEs; those with 3 or more ACEs, on the other hand, had a significantly lower average CAMS-R score of 32.5 [55]. Likewise, Voith and colleagues utilized a sample of 67 predominantly low-income men of color and used the Revised Mindfulness Self-Efficacy Scale, which encompasses facets such as emotion regulation, equanimity, and distress tolerance, to measure mindfulness. They found that ACEs were negatively correlated with mindfulness self-efficacy (*r* = −0.41) [56]. Whitaker and colleagues used 2160 Pennsylvania Head Start staff to assess the effects of ACEs and dispositional mindfulness, a trait that allows one to be aware of the present moment, on health outcomes. The results of logistic regression analysis indicated that the likelihood of having multiple health conditions, poor health behavior, and poor health-related quality of life increased with the level of exposure to ACEs. However, these same health outcomes were less common among those who reported higher levels of mindfulness. Although the significant interaction effects of ACEs and mindfulness on health outcomes from this cross-sectional analysis may not provide enough evidence to conclude causal relations among ACEs, mindfulness, and health outcomes, the results suggest that mindfulness may be a potential mediator between ACEs and health outcomes [59]. Bret and colleagues studied 385 undergraduate students and examined whether mindfulness is a mediator between ACEs and alcohol use and consequences. They used the Five Facet Mindfulness Questionnaire (FFMQ) to measure mindfulness. The FFMQ assesses five facets of mindfulness: acting with awareness, nonjudging, nonreactivity, observing, and describing. It covers both mindful attention and metacognition. The results indicated that mindfulness fully mediated the association between ACEs and alcohol use and partially mediated the association between ACEs and alcohol use consequences [57]. Likewise, using 470 adolescent students, Emirtekin and colleagues investigated the role of mindfulness, measured by the Mindful Attention Awareness Scale (MAAS), in the relation between ACEs and cyberbullying. MAAS assesses dispositional mindfulness, or open and receptive awareness of and attention to what is taking place in the present moment. The results showed that mindfulness acts as a partial mediator of the association between ACEs and cyberbullying [58]. Different measures have been used to assess mindfulness, with some focusing on dispositional mindfulness (e.g., MAAS and CAMS-R), while others offering more comprehensive measures that include both mindful attention and metacognition (e.g., FFMQ) [55,56,57]. The high correlation between mindful attention and metacognition (*r* = 0.44, *p* < 0.001) [42] increases confidence of the above assessments as measures of mindfulness.

### 2.4. Hypotheses

**Figure 1:**
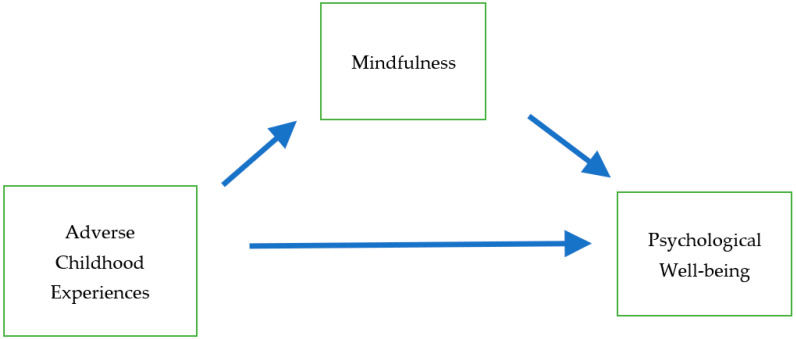
Conceptual model of adverse childhood experiences, mindfulness, and psychological well-being.

Trauma theory posits that traumatic experiences such as ACEs can impede individuals’ mindfulness and PWB and that mindfulness is a potential mediator between ACEs and PWB. Based on the above conceptual framework and existing literature, a conceptual model involving ACEs, mindfulness, and PWB is proposed, as shown in Figure 1, to examine the mediational pathway between ACEs and PWB. Specifically, we hypothesize that:

**Hypothesis 1** **(H1).** *ACEs are negatively associated with mindfulness in Chinese college students.*

**Hypothesis 2** **(H2).** *ACEs are negatively associated with PWB in Chinese college students.*

**Hypothesis 3** **(H3).** *Mindfulness is positively related to PWB in Chinese college students.*

**Hypothesis 4** **(H4).** *The effect of ACEs on PWB is mediated by mindfulness in Chinese college students.*

## 3. Data and Method

### 3.1. Sample and Procedure

The data for the present study came from an online anonymous survey taken by a sample of junior and senior students from 12 universities in China. The sampling procedure was designed to have reach a large, geographically diverse sample that would be sufficient to conduct multivariate analysis. The target size was 1000 students. We selected 12 universities, located across the north, east, south, west, and middle regions of China, to sample from in order to ensure a diverse sample spanning the country. Once universities were selected, we reached out to departments of social science, yielding a sampling frame of 2229 students. We invited students to participate based on an incentive of 10 RMB for participation (2 USD) in late September 2020. Reminders about survey completion were sent 3 and 7 days later. Prior to beginning the survey, students were informed of their voluntary participation and their ability to discontinue the survey at any time. They were also informed that their survey would be kept anonymous, with no personal information collected, and would have no bearing on their academic standing. The survey contained 25 personal and family background questions, as well as 6 scales that designed to measure ACEs, mindfulness, PWB, and other concepts. Students, on average, took 15 min to complete the survey. 1881 students participated in the online survey by early October 2020. Ten students with incomplete answers were omitted from the analytic sample, leaving us with a final sample of 1871 college students. The response rate was 80%. The research protocol was approved by the Institutional Review Board at one of the co-authors’ university (protocol number RUPro2020000301).

### 3.2. Measures

Psychological well-being was measured by the shortened 18-item version of the Psychological Well-being Scale [19]. The scale assessed six aspects of well-being: autonomy, environmental mastery, personal growth, positive relations with others, purpose in life, and self-acceptance. Each aspect was measured using three items. Example questions include “The demands of everyday life often get me down;” and “People would describe me as a giving person, willing to share my time with others.” Respondents were asked to rate how strongly they agree or disagree with the statements using a 7-point scale (1 = strongly agree; 7 = strongly disagree). The English version of the scale was translated into Chinese by two bilingual social work students and the translation was then verified by one bilingual social work faculty. We reverse-coded opposite items so that higher scores indicated greater well-being. The scores of the subscales and the total score were calculated by summing up related items. Each subscale has a range of 3 to 21, and the total score of the whole scale ranged from 18 to 126. The Cronbach’s alpha of the scale was 0.88 in this study.

Mindfulness was assessed by a 15-item Mindful Attention Awareness Scale (MAAS). MAAS was designed to measure mindfulness, a receptive state of mind, during which an individual observes what is taking place. MAAS is related to a variety of emotion regulation, behavior regulation, mental health, and well-being phenomena [60]. Past studies have shown that the Chinese version of MAAS is both valid and reliable for use with Chinese populations [51,61]. The 15 items asked participants to identify the frequency at which they experience feelings, behaviors, or mindful thoughts over the past four weeks. Examples of items include: “I rush through activities without being really attentive to them;” “I find myself doing things without paying attention;” and “I break or spill things because of carelessness, not paying attention, or thinking of something else.” The score for each item ranges from 1 to 6 (almost never to almost always). We reversed the scores so that higher scores indicated higher levels of mindfulness. The total of all scores provided ranged from 15 to 90, and the Cronbach’s alpha was 0.90 in this study.

Adverse childhood experiences during the respondent’s first 18 years of life were measured by the Adverse Childhood Experience scale (ACE). Ten items were used to measure the ACEs across three dimensions [33]: abuse (3 items), neglect (2 items), and household challenges (5 items). The Cronbach’s alpha was 0.69 for the above 10 items in this study. Example questions included “Did a parent or other adult in the household often: swear at you, insult you, put you down, or humiliate you?”, “Did you often feel that: No one in your family loved you or thought you were important or special?”, and “Did you live with anyone who was a problem drinker or alcoholic or who used street drugs?” Each affirmative answer was assigned one point. The sum of all affirmative answers represents the ACEs score. A higher score indicates a higher frequency of experiencing adverse events in the first 18 years of life.

### 3.3. Analytical Strategy

Descriptive and Pearson’s correlation analyses were first undertaken to observe the sample characteristics and the correlations among all variables. Then, we conducted Structural Equation Modeling (SEM) analysis, with a bootstrapping approach of 1000 iterations, to examine the relation between ACEs, mindfulness, and PWB. SEM, differing from regression techniques, allows simultaneous examination of direct and indirect effects through mediating variables [62]. STATA software 16.0 was used for all analyses. In results not shown, we conducted regression analyses with extensive covariates, including personal and family characteristics, the results of which indicated that the relations among ACE, mindfulness, and PWB were similar with those reported here.

## 4. Results

### 4.1. Descriptive Statistics

Table 1 presents the descriptive statistics and correlations of the variables. The average scores of PWB and mindfulness were 81.75 and 59.61, respectively, or around 4.54 per item on the 1–7 scale of PWB and 3.97 per item on the 1–6 scale of mindfulness. The sample ACE average was 0.69. Overall, the descriptive statistics suggest that the sampled college students had higher than average mindfulness and PWB, while their ACEs were low. About two-thirds of the students were female, and the mean age of the sample was 20.62. The Pearson’s correlation analyses indicated that ACEs are negatively associated with mindfulness (r = –0.22, *p* < 0.001) and PWB (r = –0.15, *p* < 0.001), supporting our first and second hypotheses. Mindfulness had a significant and positive correlation with PWB (r = 0.44, *p* < 0.001), which supports our third hypothesis. Age was positively related to mindfulness (r = 0.06, *p* < 0.05) while gender was related to age (r = −0.12, *p* < 0.001).

### 4.2. SEM Analyses

**Figure 2:**
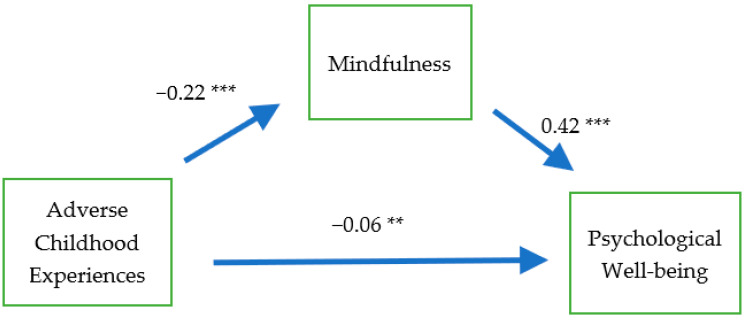
Estimated results of structural equation modeling; N = 1872. ** *p* < 0.01, *** *p* < 0.001.

We used SEM to test our conceptual model. Parameter estimates of the direct and indirect effects were determined using the bootstrapping method. Figure 2 presents the standardized coefficients of the model. Consistent with our hypotheses, ACEs were negatively associated with mindfulness (β = −0.22, *p* < 0.001), and mindfulness had a direct and positive association with PWB (β = 0.42, *p* < 0.001). ACEs were also negatively associated with PWB (β = −0.06, *p* < 0.01). The indirect effect of ACEs on PWB was negative and significant (β = −0.09, *p* < 0.001). As a result, the total effect of ACEs on PWB was −0.15 (*p* < 0.001). That is, the findings suggest that mindfulness partially mediated the association between ACEs and PWB. Mindfulness mediated 0.60 (−0.09/−0.15) of the total effects. The SEM results provide support for our fourth hypothesis, which posited that mindfulness mediates the association between ACEs and PWB.

## 5. Discussion

Empirical evidence from studies using primarily Western samples has shown that ACEs are traumatic events that can have profound consequences on health and well-being throughout one’s lifetime [8,10,43]. Research also shows that mindfulness can improve emotion and self-regulation so that individuals can be more capable of recognizing, managing, and resolving emotions and life problems, as well as making decisions that are beneficial to PWB [7,12,31,53]. However, studies have not investigated whether mindfulness mediates the association between ACEs and PWB in college students, especially in non-Western samples. The findings from SEM analysis in this study indicate significant and negative effects of ACEs on mindfulness and PWB of Chinese college students. Mindfulness showed strong and positive effects on PWB, whereby increasing one SD of mindfulness was associated with about a 0.42 SD increase in PWB. Mindfulness also partially mediated the effects of ACEs on PWB. Chinese college students with ACEs thus experience lower PWB due, in part, to decreased mindfulness. Mindfulness being strongly associated with self-regulation [31], the above findings are consistent with past studies, which have largely used Western and non-college samples, that have suggested that those with trauma histories experience emotional and behavioral dysregulation [43] and lower mindfulness [54,55]. This, in turn, can lead to poor PWB, including severe psychopathology [8,46]. The negative effects of ACEs on mindfulness and PWB appear to persist across cultures, populations, and age groups.

The findings of this study have practice and research implications. With respect to practice, given the strong estimates of mindfulness on PWB, this study points to mindfulness as a key point of intervention for individuals who have a history of ACEs. This underscores the importance of mindfulness interventions in potentially buffering the effects of ACEs on PWB. Empirical studies have shown that mindfulness-based stress reduction (MBSR), mindfulness-based cognitive therapy (MBCT), and mindfulness-based interventions (MBI) all can effectively reduce psychological distress and promote mental health and PWB [63,64,65,66]. MBSR, a group program developed by Kabat-Zinn in the 1970s, is composed of two main components: mindfulness meditation and yoga. The program, although a flexible and customizable approach to stress reduction, requires significant time commitment. The typical MBSR program requires participants to allocate 45 min a day, six days a week for 8 weeks. The main elements of MBSR includes mindful attention, attitude (e.g., wisdom, compassion, and peace of mind), and intention (e.g., self-regulation, self-exploration, and self-liberation). MBCT, an adaptation of MBSR, uses a similar format and structure but explicitly focuses on cognitive therapy to help people who suffer repeated bouts of depression and chronic unhappiness. MBI, also based on MBSR, has fewer elements and requires less of a time commitment [65,66,67].

Counseling professionals on college campuses can design various mindfulness-based programs to assist students with ACEs, depending on students’ clinical presentation and program resources. Brief MBIs implemented with college student samples, including healthy students [68] and those classified as binge-drinkers [69], have been shown to increase mindful awareness, self-control [68], and dispositional mindfulness [69]. These interventions were also able to decrease psychological distress [68], stress, anxiety, depression [70], and binge-drinking [69]. Past cross-cultural research has also supported the efficacy of mindfulness interventions with children [48], adolescents [51], and emerging adults [71] in China, highlighting the potential of implementing such interventions in the Chinese college student population. It is worth noting that intervention curricula should cover comprehensive dimensions of mindfulness, including intention, attention, and attitude, to better promote emotional regulation, mindful attention, and decentering [31,45,46,47]. Given that the ACE questionnaire assesses ACEs during the respondent’s first 18 years of life, the immediate time to buffer the negative effects of ACEs on PWB is during emerging adulthood, between the ages of 18 and 25 [14,15]. In addition, the college years have been found to be a critical phase of development [14,15,16,17,18,19]. Positive development in emerging adulthood is essential given that this period tends to be characterized by peak levels of risk-taking behaviors [72], which can be exacerbated by ACE history [10]. Thus, the findings of this study provide a strong base for colleges to implement MBIs for at-risk students in China and beyond.

The findings of this study also have implications for future research. These initial results provide support for further examination of the relations among ACEs, mindfulness, and PWB, as all three concepts contain multiple dimensions and may be measured and operationalized differently. ACEs include three dimensions of life experience (abuse, neglect, and household challenges), so future studies can examine the extent to which each occurrence, along with cumulative occurrences, affects mindfulness and PWB. Likewise, PWB covers multiple dimensions, including self-acceptance, positive relationships with others, autonomy, environmental mastery, purpose in life, and personal growth; it is likely that ACEs and mindfulness may differentially affect each PWB dimension. Measures of mindfulness should cover comprehensive dimensions of mindfulness as well. The measurement instrument used to assess mindfulness in this study, MAAS, was designed to measure the receptive state of mind [62]. Although MAAS has been found to be positively related to emotion regulation, behavior regulation, mental health, and well-being [60], and although there is a high correlation between mindful attention and metacognition [42], future studies can consider using a comprehensive measure of mindfulness, such as FFMQ, which includes facets like non-judgmental inner experience and non-reactivity [73].

This study has several limitations. First, our analyses were based on a cross-sectional dataset, which can only approximate an associative relationship, rather than a causal one, among volunteering, volunteer motivation, and PWB. Future research can use a longitudinal design to examine the causal relationship of these variables. Second, there were other unobserved variables that could affect PWB but were not included in the study, such as personality traits, peer support, and academic stressors. The absence of these unobserved variables may have effects on the estimates reported in this study. Third, data gathered on key variables such as ACEs, mindfulness, and PWB were from self-reports of the subjects. Self-reporting leaves our data subject to unintended and intended reporting errors, including social desirability bias. Future studies might consider triangulating findings from different data sources, such as peer or teacher reports. Fourth, the findings of this study are based on data from the social science departments of 12 colleges across China. Although the sample size and diversity of colleges across regions both increase our confidence, the extent to which these findings can be generalizable to all Chinese college students is unknown and requires further research.

## 6. Conclusions

This study used 1871 college students across China to investigate the roles of ACEs on mindfulness and PWB and whether the effect of ACEs on PWB was mediated by mindfulness. The findings show that ACEs had negative effects on mindfulness and PWB. Further, mindfulness partially mediated the effects of ACEs on PWB in Chinese college students. Despite some of the limitations mentioned above, the present study contributes to knowledge on the role of mindfulness in the association between ACEs and PWB in Chinese college students, calling for the need to encourage mindfulness interventions particularly among vulnerable students, such as those with a history of trauma, to buffer the deleterious effects of ACEs on PWB.

## Figures and Tables

**Table 1:** Descriptive statistics and correlations of key variables.

	Mean (S.D.)	1	2	3
1. Psychological Wellbeing [24–121]	81.75 (12.29)	---		
2. Mindfulness [15–90]	59.61 (10.84)	0.44 ***	---	
3. Adverse Childhood Experiences [0–10]	0.69 (1.28)	−0.15 ***	−0.22 ***	---

Note: N = 1871. Numbers in parentheses in Column 1 show observed ranges of the variables. *** *p* < 0.001.

## Data Availability

The data presented in this study are available on request from the corresponding author.
